# Approaches for Total Hip Arthroplasty: A Systematic Review

**DOI:** 10.7759/cureus.34829

**Published:** 2023-02-10

**Authors:** Niketa Patel, Paresh Golwala

**Affiliations:** 1 Department of Physiotherapy, Sumandeep Vidyapeeth Deemed to be University, Vadodara, IND; 2 Department of Orthopedics, Sumandeep Vidyapeeth Deemed to be University, Vadodara, IND

**Keywords:** anterolateral approach, posterolateral approach, lateral approach, posterior approach, anterior approach, surgical approach, total hip replacement, total hip arthroplasty

## Abstract

These surgical procedures have their own challenges, limitations, and success rate. The choice of surgical approach will depend on many factors including the surgeon’s choice, type of pathology, bone stock, age of the patient, and experience of the surgeon. Whichever approach is used for total hip arthroplasty (THA), the primary goals of the surgery would be pain relief and enhancement in the quality of the patient’s life suffering from hip pathologies. To further understand the advantages and potential pitfalls associated with different surgical approaches, we conducted a review study comparing different surgical approaches for THA in terms of their clinical and functional outcomes. All the studies done on surgical approaches used in THA published articles in the English language and from 2015 onward were included in the review. The databases searched were COCHRANE, MEDLINE, PEDRO, CINHAL, etc. Search engines that were searched were Google Scholar, Pub Med, and Science Direct. As per the inclusion criteria, out of 50 studies, 26 studies were included in the study which underwent critical analysis. Considering all the factors reviewed from the literature, the posterior approach or posterolateral approach is optimally beneficial.

## Introduction and background

In the last 40 years, total hip arthroplasty (THA) has topped the list of most successful interventions in the orthopedic fraternity. The lifestyle in western countries along with the increase in the incidence of obesity, over the past few decades, the number of THA surgeries has started expanding. THA is no more restricted to the elderly population, but it has also shown successful results in the younger populations who are still working and are not retired. Early discharge, less use of analgesics, cost-effectiveness, early functional recovery, and shorter immobilization are a few demands by the patients. Such demands have led to the development of alternative surgical procedures which aims to improve the success of the THA [1].

These surgical procedures have their own challenges, limitations, and success rate [2]. The choice of surgical approach (SA) will depend on many factors including the surgeon’s choice, type of pathology, bone stock, age of the patient, and experience of the surgeon [2,3]. Whichever approach is used for THA, the primary goals of the surgery would be pain relief and enhancement in the patient’s quality of life suffering from hip pathologies [4,5]. As there exists many SA for THA, there also exists a thrust to know which approach is better than the other and in which manner. Since, the procedures differ in the incision, surgical planes, and technique used, it is important to consider how various approaches can affect the surgical success rates. The four most popular strategies are anterolateral (or Watson-Jones), direct lateral (or Hardinge), direct anterior (or Heuter), and posterior (or Moore) [5,6].

The posterior technique has been associated with a higher risk of postoperative displacement because it disrupts the posterior joint capsule, whereas the direct lateral approach (DLA) has been linked to an increased risk of superior gluteal nerve injury, post-operative abductor weakness, and limping due to abductor muscle disruption. Due to prolonged anterior retraction, it has been postulated that the anterolateral approach (ALA) damages the superior gluteal nerve. The direct anterior approach (DAA) has also been linked to more serious wound problems [6]. To further understand the advantages and potential pitfalls associated with different surgical approaches, we conducted a review study comparing different surgical approaches for THA in terms of their clinical and functional outcomes.

## Review

Methodology

This review has focused on the advantages and pitfalls of different approaches in a surgery used in THA. The inclusion criteria involved studies done on surgical approaches used in THA-published articles in the English language and articles published from 2015 onwards. A comprehensive literature search was undertaken using PRISMA-S guidelines in major health databases and search engines. The databases searched were COCHRANE, MEDLINE, PEDRO, and CINHAL. Search engines that were searched were Google Scholar, PubMed, and Science Direct. For a targeted search, the keywords like THA, Total Hip Replacement (THR), surgical approach, anterior approach, posterior approach (PA), lateral approach (LA), posterolateral approach (PLA), and ALA were used. The data were searched from electronic sources that involved databases, electronic libraries, journals, and google scholar. The inclusion criteria involved studies done on surgical approaches used in THA published articles in the English language, and articles published from 2015 onwards following this, the included articles were critically appraised. The description, analysis, and quality, of the extracted data along with an analysis of the results and their interpretation will be performed. The purpose was to combine the information from various sources to fulfill the aim of the study.

Results

**Figure 1:**
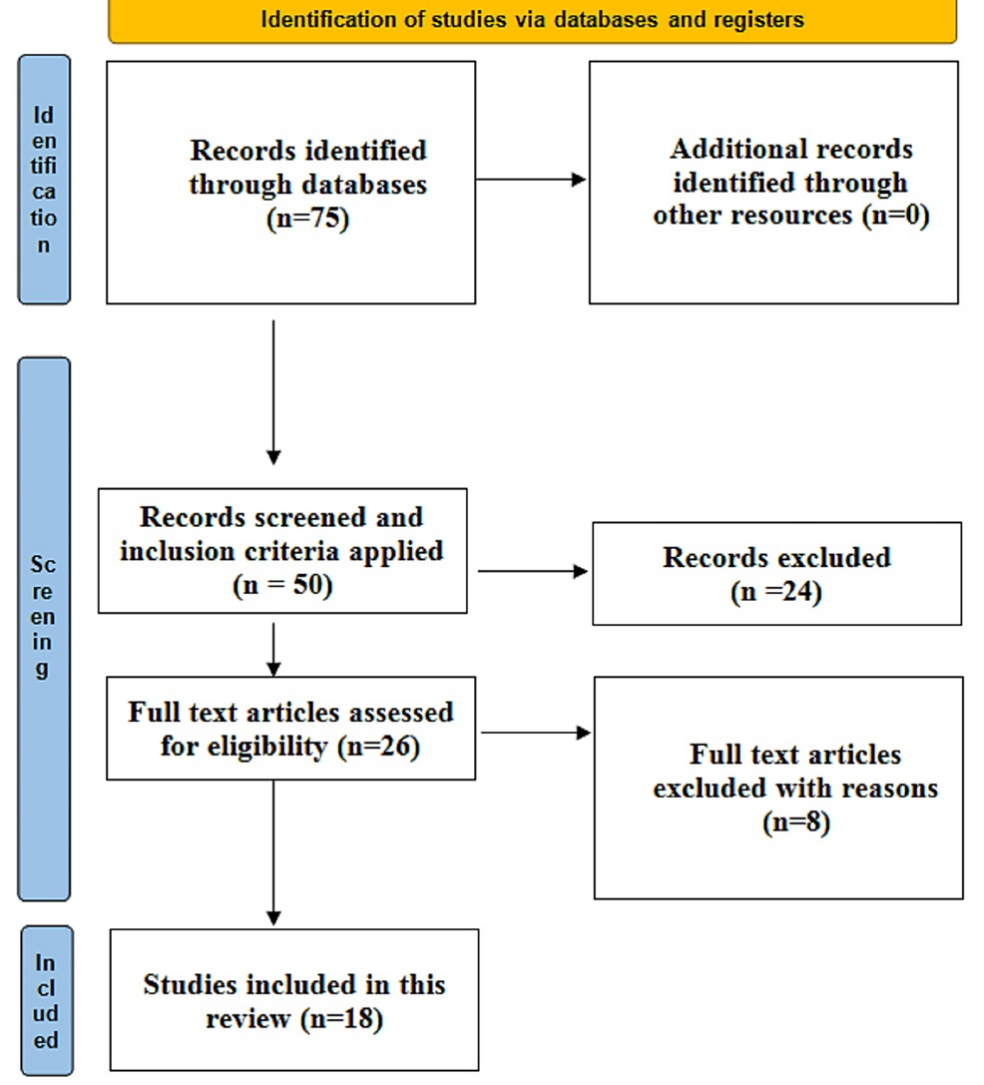
PRISMA-S flowchart describing search strategy.

Overall, 75 papers consisting of surgical approaches for THA were downloaded. Of these 75 articles, 50 articles underwent full-text screening (24 studies were excluded as they did not mention surgical approaches). As per the inclusion criteria, out of 50 studies, 26 studies were included in the study (Figure 1, Table 1).

**Table 1:** Number of patients operated using different surgical approaches for hip surgeries.

		Number of patients as per the surgical approaches					
SR.NO.	TITLE	ANTERIOR	ANTEROLATERAL	POSTERIOR	POSTEROLATERAL	LATERAL	NORTHERN
1	Anterior Approach in Total Hip Replacement [7].	90	--	--	--	--	--
2	Do Postoperative Results Differ in a Randomized Trial Between a Direct Anterior and a Direct Lateral Approach in THA? [8].	84	--	--	--	80	--
3	A prospective randomized comparison of the minimally invasive direct anterior and the transgluteal appro ach for primary total hip arthroplasty [9]	73	--	--	--	50	--
4	Direct anterior versus lateral approaches for clinical outcomes after total hip arthroplasty: a meta-analysis [10]	236	--	--	--	239	--
5	A Prospective randomized clinical trial in total Hip Arthroplasty - Comparing early results between the Direct Anterior Approach and The Posterior Approach [11].	37	--	38	--	--	--
6	Does Surgical Approach Affect Outcomes in Total Hip Arthroplasty Through 90 Days of Follow-Up? A Systematic review with Meta analysis [12].	524	--	520	--	--	--
7	Are there more wound complications or infections with direct anterior approach total hip arthroplasty? [13].	399	--	796	--	--	--
8	Influence of surgical approach on complication risk in primary total hip arthroplasty Systematic review and meta-analysis [14].	21,013	--	143,294	--	--	--
9	Early Rate of Revision of Total Hip Arthroplasty Related to Surgical Approach An Analysis of 122,345 Primary Total Hip Arthroplasties [15]	32,086	--	65,791	--	24,468	--
10	Posterolateral versus direct anterior approach in total hip arthroplasty (POLADA trial). A randomised controlled trial to assess differences in serum markers [1]	23	--	--	23	--	--
11	High risk of positive Trendelenburg test after using the direct lateral approach to the hip compared with the anterolateral approach A SINGLE-CENTRE, RANDOMIZED TRIAL IN PATIENTS WITH FEMORAL NECK FRACTURE [16].	--	75	--	--	75	--
12	Effect of femoral head size and surgical approach on risk of revision for dislocation after total hip arthroplasty An analysis of 166,231 procedures in the Dutch Arthroplasty Register (LROI) [17].	14,446	12,744	--	100,823	35,830	--
13	Total hip arthroplasty using a posterior minimally invasive approach – results after six years [18].	--	--	103	--	121	--
14	Contemporary posterior surgical approach in total hip replacement: still more reoperations due to dislocation compared with direct lateral approach? An observational study of the Swedish Hip Arthroplasty Register including 156,979 hips [19].	--	--	89,539	--	67,440	--
15	Retraction: Post-operative neuropathy after total hip arthroplasty [20].	--	--	39056	--	--	--
16	Hospitalization length, surgical duration, and blood lost among the approaches for total hip arthroplasty: a Bayesian network meta‑analysis [21].	1,310	320	1,310	392	932	--
17	Surgical approach significantly affects the complication rates associated with total hip arthroplasty [22].	1,329	30	1,657	--	393	165
18	Comparison of intra and post-operative complication rates among surgical approaches in Total Hip Arthroplasty: A systematic review and meta-analysis [6]	30,101	23,603	185,931	--	57,446	--
TOTAL		101,755	36,772	528,035	101,238	186,681	165
9,54,646		ANTERIOR	ANTEROLATERAL	POSTERIOR	POSTEROLATERAL	LATERAL	NORTHERN

Discussion

After going through all the articles mentioned earlier, the total number of patients was 954,646 operated for hip surgeries. Of these, AP was performed on 101,755 (10.65%) patients, ALA was performed on 36,772 (3.85%) patients, PA was performed on 528,035 (55.31%) patients, PLA was performed on 101,238 (10.60%) patients, LA was performed on 186,681 (19.55%) patients and 165 (0.04) patients were operated with Northern approach.

Ease of approach

The surgeon mostly practices the approach which he learned during his PG days. They are more comfortable and confident in the skills that they practiced during their resident ship. The literature pooled from prospective randomized clinical trials (RCT) [11] and a systematic review (SR) and meta-analysis (MA) [12] revealed that the decision of SA should be dependent on the opinion, and experience of the surgeon, and current hip joint conditions.

The overall analysis shows that the PA (i.e., 55.31% of 9,54,646 patients) and LA (i.e., 19.55% of 9,54,646 patients) are the choices of SA by the surgeon. Also, Skoogh et al. [19], in their study, observed that the most common SA for THA is DLA and PA. So, AA is preferred by only limited surgeons (3.85%).

Risk of infection

THR has evolved to its optimum best at present. It has the latest implant designs, a new SA with the goal to have minimal soft tissue injuries, updated equipment, and Operation Theaters. According to an SR and MA conducted by Miller et al., the risk of infection is relatively low (RR=0.55) in the DAA compared to PA in primary THA when they compared complications in DAA (21,013 patients, 13%) with PA (143,294 patients, 87%) with at least one year mean follow up [14].

We found another similar study by Tissot et al. which determined to find whether the type of SA plays a role in the rate of complications and infections after surgery. For this, they compared DAA (399 patients, 33%) and Direct PA (796 patients, 67%) in patients who operated for THR. It was observed that the rate of infection in the DAA group was higher (1.5%) than in the PA group (0.75%) [13]. A higher rate of infection with AA may be due to obesity, obesity is the known risk factor for infection.

Soft tissue damage

Minimal tissue damage is one of the important goals in THR surgery which reflects positively in the rehabilitation and the functional outcome in the THR patients and their quality of life. In a study AA (90 patients) was appreciated as having minimal soft tissue dissection which resulted in early mobilization and early discharge from the hospital [7]. On the contrary, a study by Rykov et al. examined the muscle damage in 46 THA patients operated either by AA or PLA [23]. They measured muscle damage using serum markers, i.e., serum creatine kinase (CK) and C-reactive protein (CRP) and found no significant relevance between the groups.

We found another study that compared ALA with DLA to measure muscle damage in patients with hemiarthroplasty [24]. They used (CK) levels to measure the damage. The study reported high postoperative CK levels in the ALA group indicating muscle damage and the presence of inflammation. Therefore, preserving surrounding soft tissues will help to achieve short-term functional outcomes and thus early discharge.

Nerve damage

Of other complications, nerve damage after THR is not unknown. One of the main complications of AA (90 patients) is lateral femoral cutaneous nerve damage (8%) [7]. We found 03 studies supporting this finding. First, Mjaaland et al. conducted a randomized study to observe the postoperative results of THA [8]. For this, they compared DAA (84 patients) with DLA (80 patients) in terms of nerve injury in THA patients. They noticed that their five patients (6%) had nerve injuries (four transient femoral nerves; one tibial nerve, and one lateral femoral cutaneous nerve) in the DAA group, of which four patients showed recovery within three months of the surgery and one patient had permanent damage to the nerve. Second, Reichert et al. conducted an RCT to compare the minimally invasive DAA (73 patients) with the transgluteal LA (50 patients) in patients undergoing THA and reported that in their study, injury to the lateral femoral cutaneous nerve is shown in the AA group patients resulting in its palsy (4.1% palsy rate) [9]. Third, in a systematic and meta-analysis, the effect of SA on the complication risks following hip surgery was reviewed between DAA (21,013 patients) and PA (143,294 patients) in primary THA with at least one-year mean follow-up for the complication risks following THR and concluded that higher rate of patient-reported nerve injury is found in DAA (RR = 2.3, p = 0.01) [14].

A study reviewing sciatic nerve injury in the patients operated on using PA for THR revealed that out of 39,056 THR patients, 93 patients (0.24%) had sciatic nerve injury [20]. They emphasized that the PA may cause sciatic nerve injury because acetabular retractors may impinge the sciatic nerve posteriorly. The study concluded that other than lengthening of the extremity and uncemented femoral implant, PA is the risk factor associated with sciatic nerve injury.

Thus, from the data pooled for the review, it is clear that there is a high risk of injury to the lateral femoral cutaneous nerve in patients operated on using the DAA and the sciatic nerve may get injured in patients operated on using PA.

Trendelenberg' sign

Trendelenburg sign is seen in weak hip abductors. There are different SA used in THR in which gluteus medius may be split or retracted or spared. It is well known that the residual effect of surgery in which the hip abductors have reinserted results in Trendelenburg gait and hence the surgeon prefers to choose a surgical approach where these muscles are minimally damaged or spared.

An RCT compared direct AA (84 patients) with DLA (80 patients) in THA patients and revealed that within 24 months of THA, patients who were operated on using DLA showed a positive Trendelenburg sign [8]. This suggests that as per the patients’ personality, if they have not regained abductor muscle strength at 24 months, they are unlikely to have normal muscle power. These findings were supported by other similar RCTs [14] which compared DAA and transgluteal LA for THA. The study resulted in one gluteal insufficiency and three limb length discrepancies. Another study evaluated Trendelenburg sign in hemiarthroplasty patients comparing the two SA - ALA and LA and observed a high risk of positive Trendelenburg sign in the DLA group [16]. A six-year follow-up study that compared PA (103 patients) and traditional LA (121 patients) in THA patients showed that the Trendelenburg sign was positive, i.e., weakness in hip abductor muscles in most of the patients who were operated on with LA whereas no signs were seen in patients operated with PA with statistically insignificant results and at the end of six years, both the groups were same in the radiologic and clinical outcomes [18]. Even Bostock et al. in their SR and MA reported that the Trendelenburg sign was more commonly seen in LA than in PA [25].

Splitting, retraction, or reinsertion of gluteus medius muscle results in weak hip abductors leading to positive Trendelenburg sign/ gait following hip surgeries. In the reviewed studies, LA was compared with different SA for the risk of the Trendelenburg sign. All the studies (approximately > 900 patients) had a similar conclusion that there are higher chances of developing positive Trendelenburg sign in patients operated with LA.

Dislocations

Though there is an increase in updated knowledge of risks factor for post-operative dislocation and the availability of advanced treatment still there are cases of dislocations following THR. We found few studies that have worked to find the impact of SA on the risk of dislocations following THR. Among those, in one of the studies involving AA in THR concluded that AA had low chances of dislocations because of minimal soft tissue dissection and low muscle detachment [7]. These findings were supported by two systematic and meta-analysis [6,14]. One study [14] stated that the risk of dislocation following THR is very low in AA (21,013) compared to PA (143,294) with at least one-year mean follow-up in primary THA. While another study [6] stated that patients who were operated on with PA (185,931) had higher chances of hip dislocations compared to AA (30,101), LA (57,446), and ALA (23,603).

We found a study favoring AA which analyzed different SA (AA, LA, and PA) for the rate of revision surgeries after THA [15]. The study revealed that revision surgeries due to dislocations and infection were very few in AA (32,086) compared to other approaches.

Our interest was aroused in a 2017 study because it analyzed 166,231 and 3,754 primary THA and subsequent revision THAs, respectively, done due to dislocations [17]. They studied the role of SA and prosthetic femoral head size on hip dislocations and stated that hip dislocation was more in PLA (100,823) whereas, AA (14,446) and ALA (12,744) showed less number of dislocations. As per the studies, strategies accepted to reduce dislocation was that 32 mm head size reduced the rate of dislocations for all the approaches. But for PLA, 36mm head size drastically reduced the chances for hip dislocation and therefore such head size should be considered for patients who are at higher risk of dislocations.

Shoogh et al. compared PA (89,539) with LA (67,440) following hip surgery to study the risk of dislocation and concluded that due to dislocation the risk of reoperation was higher in PA within two years of THR [19]. Contrary to the above study we found a study by Berstock et al., which found on the basis of 517 patients that dislocations were seen more commonly in LA than in PA [25].

Blood loss

There is extensive bleeding during arthroplasty surgeries and thus need for blood transfusion is in high demand. Blood transfusion carries the risk of early morbidity and mortality. Wang et al. conducted a meta-analysis to study the effect of SA on blood loss during hip surgeries [10]. They compared DAA (236 patients) with LA (239 patients) and reported that the DAA group had less blood loss and thus has an important role in reducing blood loss. The reason behind this may be the DAA is an intramuscular and internervous approach.

We found another study that measured blood loss during hip surgeries. It reported that in their study, the ALA group (320 patients) had the lowest result in blood loss (SMD: 61.20; 95% CI), and the PLA group (392 patients) had the highest result in blood loss (SMD: 433.50; 95% CI) [21]. These findings were congruous to the findings of Aggarwal et al. who stated that in their study, the ALA group had the lowest estimated blood loss (270.3 mL) [22]. Hence, from the data reviewed, it can be stated that AA and ALA have an advantage in reducing blood loss in THR.

Bone loss

Studies have shown that bone loss occurs at the proximal femur/greater trochanter following THR. This results in an increase in fracture risk around the implant. A study assessed the effect of ALA and DLA on periprosthetic bone loss in patients operated on for THR following neck femur fracture [26]. They measured bone health with the help of BMD and DXT. The study reported that at 3 months following THR, there was a significant bone loss in the proximal gruens regions in patients operated on using DLA (6.5% of bone loss) than in the ALA group (1.6% of bone loss) which increased at 6 months to 3.3% of bone loss in ALA and 8.1% of bone loss in DLA. Hence, it can be concluded that SA especially DLA does have an influence on the periprosthetic bone loss.

Revision surgeries ratio 

Revision surgeries are the key concern in the world of hip surgeries. It not only delays achieving the functional goal but also affects the patient psychologically as well as economically and is the most common reason for revision surgery as presented by Zijlstra et al. in their study [17]. They stated that the most common reasons for revision surgeries from the 166,231 patients’ databases were implant loosening, dislocations, periprosthetic fracture, and infection. They analyzed that the risk of revision surgery for dislocation after THR was higher in PLA than in AA, ALA, and LA, immaterial of the head size. Whereas the risk of revision surgery due to periprosthetic fracture or femoral implant loosening was higher in AA and ALA. The author also specified that a 36mm head size had a significantly higher risk of revision surgery due to periprosthetic fracture and implant loosening.

Hoskins et al. studied the relation between SA and the early rate of revision of THR in 122,345 primary THR [15]. They reported that the AA (32,086, 26%) was associated with increased rates of revision for periprosthetic fracture and component loosening whereas LA (24,468 patients, 20%) and PA (65,791 patients, 54%) were linked to dislocation and infection after hip surgeries.

Revision surgeries were required for various causes like dislocations, infection, bone resorption, implant loosening, and periprosthetic fracture. Some of the indications are leading to revision after a long period and as such the approaches cannot be the sole cause for the revision surgeries.

## Conclusions

Even though the PA is the most widely used and preferred method, all other methods fall short when taking into account characteristics like ease of access, infection rate, bone loss, etc. Only a small number of surgeons use the DAA, and infection is one of the most frequent side effects, along with increased bone resorption. Although all kinds of LAs (conventional/direct) are appropriate, Trendelenburg gait is more common in patients. It is best to take a posterior or PLA while taking into account all the aforementioned criteria.
